# Comprehensive genomic characterization of programmed cell death-related genes to predict drug resistance and prognosis for patients with multiple myeloma

**DOI:** 10.18632/aging.206234

**Published:** 2025-04-01

**Authors:** Yan Li, Fuxu Wang, Hongbo Zhao, Zhenwei Jia, Xiaoyan Liu, Guirong Cui, Tiejun Qin, Xiaoyang Kong

**Affiliations:** 1 Hematology Department, Handan First Hospital, Handan 056001, China; 2 Department of Hematology, Key Laboratory of Hematology of Hebei Province, Second Hospital of Hebei Medical University, Shijiazhuang 050000, China; 3 MDS and MPN Centre, Institute of Haematology and Blood Diseases Hospital, Tianjin 300020, China

**Keywords:** multiple myeloma, programmed cell death, molecular subtypes, immune status, drug sensitivity

## Abstract

Background: Multiple myeloma (MM) is a cancer that is difficult to be diagnosed and treated. This study aimed to identify programmed cell death (PCD)-related molecular subtypes of MM and to assess their impact on patients’ prognosis, immune status, and drug sensitivity.

Methods: We used the ConsensusClusterPlus method to classify molecular subtypes with prognostically relevant PCD genes from the MM patients screened. A prognostic model and a nomogram were established applying one-way COX regression analysis and LASSO Cox regression analysis. MM patients’ sensitivity to chemotherapeutic agents was predicted for at-risk populations.

Results: Six molecular subtypes were classified employing PCD-related genes, notably, three of them had a higher tendency for immune escape and two of them were correlated with a worse prognosis of MM. Furthermore, the C3 subtype had activated pathways such as oxidative phosphorylation and DNA repair, while the C2 and C4 subtypes had activated pathways related to apoptosis. The Risk score showed that the nomogram can correctly predict the OS for MM patients, in particular, patients in the high-risk group had low overall survival (OS). Pharmacovigilance analyses revealed that patients in the high-risk and low-risk groups had greater IC_50_ values for the drugs SB505124_1194 and AZD7762_1022, respectively.

Conclusions: A 12-gene Risk score model developed with PCD-related genes can accurately predict the survival for MM patients. Our study provided potential targets and strategies for individualized treatment of MM.

## INTRODUCTION

Multiple myeloma (MM) is the second most frequent hematological tumor [1–3]. Around 10% of all hematological malignancies are MM cases, with the majority of patients over 40 years of age and a 5-year survival rate of 53% [4, 5]. Multifocal spread throughout the bone marrow without apparent clinical symptoms is the main characteristic of early-stage MM. In the last ten years, the advent of immunomodulatory medications, proteasome inhibitors, and combination therapy has improved the treatment of MM. For instance, immunomodulator drugs (IMiDs) are considered as a single-agent maintenance therapy after autologous stem cell transplantation and can be used in combination therapy for all stages of MM [6]. Daratumumab, the first CD38 monoclonal antibody medication authorized for the treatment of MM patients, has shown promising effect on treating both newly diagnosed MM and relapsed or refractory MM [7]. Nevertheless, MM is still largely incurable and many patients relapse due to immune evasion and therapeutic resistance [8, 9], necessitating the discovery of novel molecular biomarkers to improve patient risk classification and therapy response prediction.

Since the primary objective of cancer treatment is to eradicate tumor cells, inducing cancer cell death has become a crucial strategy [10]. The active process of programmed cell death (PCD) preserves bodily growth and survival [10], while non-PCD is a highly structured process that typically involves the regulation of gene expression and signal transmission [11]. In contrast, PCD is a type of necrosis stimulated by trauma, infection, or ischemia [12]. Although the morphology, biochemistry, and signaling pathways of different forms of cell death vary, they are all actively executed by cells as a part of PCD process, which is essential for maintaining tissue homeostasis and supporting the immune system [13, 14]. Moreover, the development of numerous diseases, including immunological problems, tissue damage, neurodegeneration as well as malignancies such as MM [15], breast cancer [12] and hepatocellular carcinoma [16], are closely linked to PCD. Furthermore, there is a strong correlation between the prognostic evaluation of cancer patients and PCD-related genes. Gu et al. discovered five important PCD genes [17] that affect hepatocellular carcinoma patients by controlling immunological function, inflammatory pathways, and treatment response. Recent evidence suggested that PCD-associated genes contribute to the prognostic prediction, immune profile, and treatment of endometrial cancer of the uterine corpus [18]. Similarly, PCD-associated traits have been identified to evaluate the immune microenvironment landscape, drug sensitivity and prognosis for patients with cutaneous melanoma [19] and acute myeloid leukemia [20]. Based on the above findings, the present work classified molecular subtypes of MM based on prognostically significant PCD-related genes by consensus clustering method, and further compared the differences between the subtypes in terms of pathway characteristics and clinical features. Differential expression analysis and LASSO were employed to determine genes related to PCD phenotype. Finally, we constructed a clinical prognostic model to offer novel understanding for the targeted drug therapy and clinical diagnosis of adjuvant MM.

## MATERIALS AND METHODS

### Data collection

The Cancer Genome Atlas (TCGA, https://portal.gdc.cancer.gov/) database was accessed to obtain the RNA-seq data, corresponding clinical characteristics, and follow-up data of 859 MM patients from the MMRF-COMPASS project. A total of 844 MM samples were obtained after data preprocessing. Then, these samples were randomly assigned into training set (592 cases) and validation set (252 cases) at the ratio of 7:3 to ensure the representativeness and randomness of the sample distribution. In addition, 55 cases of MM samples in the GSE57317 dataset were collected from the Gene Expression Omnibus (GEO, https://www.ncbi.nlm.nih.gov/geo/) database. Importantly, genes related to PCD were obtained from a previous study [12].

### Preprocessing of RNA-seq data for TCGA

1) Eliminating samples without clinical follow-up data;

2) Retaining samples with a survival time longer than or equal to thirty days;

3) Eliminating samples without survival state;

4) Gene Symbol Conversion from Ensembl;

5) The median was used to normalize the gene with numerous gene symbols.

### GEO data preprocessing

After downloading the standardized microarray probe expression data (GSE57317), the probe expression was transformed into gene expression using the platform annotation file. The average expression of several probes corresponding to the same gene was used as the expression value of the gene, while the probes were eliminated when only one probe matched several genes. The maximum expression value was taken when more than one probe matched to the same gene. MM specimens were removed, and patients in good survival status with a survival time longer than 30 days were included in this analysis. Finally, 55 MM samples in GSE57317 were kept.

### Identification of molecular subtypes using PCD-related genes

Unsupervised clustering is a data mining approach that uses only internal attributes to identify unknown clusters of potential objects. Consensus clustering (CC) uses repeated subsampling and clustering to produce quantitative and graphical “stability” proof. Specifically, ConsensusClusterPlus extends the CC algorithm by initially subsampling a specific percentage of items and a specific percentage of features in the data matrix. A user-specified clustering method then splits each subsample into several categories [21]. This study applied CC to classify the molecular subtypes in the “ConsensusClusterPlus” R program [21]. The 500 bootstraps were conducted utilizing the “km” algorithm and “1 - Spearman correlation” as a metric distance. Each bootstrap included 80% of the training set of patients. The optimal subtype was determined by consistent cumulative distribution function, and the number of clusters was between two and ten.

### Association between molecular subtypes and immune properties

The correlation between immune checkpoint genes in different molecular subtypes was analyzed. A total of 67 immune checkpoint genes taken from early research were included as the representative immune checkpoints [22]. Using Kruskal, the levels of immune checkpoint gene expression in the molecular subtypes were assessed. Tumor immune dysfunction and exclusion (TIDE) algorithm was employed to evaluate potential responses of MM patients to immune checkpoint blockade (ICB) [23]. Specifically, differences in T-cell rejection characteristics in different MM molecular subtypes were further compared based on the gene expression profiles of dysfunction, exclusion, tumor-associated macrophage M2 types (TAM.M2), myeloid-derived suppressor cell (MDSC), and cancer-associated fibroblast (CAF) using TIDE software [23, 24].

### Functional enrichment analysis

Single-sample GSEA (ssGSEA) [25] refers to the absolute enrichment of a gene set in each sample from a dataset. Here, the R package “GSVA” [26] was used to calculate the scores of the 34 biological pathways to obtain ssGSEA scores for each sample. Furthermore, based on the HALLMARK gene set in the Molecular Signatures Database (MSigDB, http://software.broadinstitute.org/gsea/msigdb) [27], we performed GSEA pathway analyses to identify unique biological processes and evaluate differentially activated pathways across the molecular subtypes.

### Development of a PCD-related prognostic model

Differentially expressed genes (DEGs) from previously identified molecular subtypes were screened by the R package “limma” [28], and those with |logfc|>log2(1.5) and *p*<0.05 in each molecular subtype were extracted. Moreover, we employed one-way Cox regression analysis to screen genes with differential expression and utilized the “survival” R package to identify multiple important PCD-related features (*p*<0.05) [29]. Next, the R package “glmnet” was utilized to further limit the number of genes, and the prognostically relevant genes were determined by LASSO regression [30]. Next, the Risk score for the MM patients was calculated by the following formula: Risk score = Σβi × Expi), where β is the gene Cox regression coefficient and i is the gene expression level. Then the Risk score was subjected to Z-score, and the MM samples of the training set, validation set, and GSE57317 dataset were classified into high-risk group (zscore >0) and low-risk group (zscore <0) by the threshold value of “0”. Prognostic analyses were performed by plotting the Kaplan-Meier (KM) survival curves, and significant differences were assessed by log-rank test. Furthermore, receiver operating characteristic (ROC) curves were plotted and 1-, 2-, and 3-year area under the curve (AUC) was computed using the R package “timeROC” [31].

### Correlation analysis between risk scores and drug sensitivity

Based on in the Genomics of Drug Sensitivity in Cancer (GDSC) database, the sensitivity of MM patients to several drugs was predicted using the “oncPredict” package [32] in R software. In addition, the IC_50_ values of drugs for samples from the MMRF-COMPASS training set cohort was also calculated. Further, the correlation between drug sensitivity and Risk score was predicted using Pearson correlation analysis, with *p*<0.05 and |cor|>0.3 being considered as statistically significant.

### Statistical analysis

R program (v4.2.1) was used to conduct statistical analysis. The Student t-test or Wilcoxon test was used to compare the two groups. The Kruskal-Wallis one-way ANOVA was applied to assess the comparisons between two or more groups. We plotted Kaplan-Meier survival curves and evaluated the variations between the curves. Correlation analysis between continuous variables was performed using Spearman’s rank correlation. *P*-value of 0.05 served as the cutoff for statistical significance in all analyses. Ns represented *p* > 0.05; *p < 0.05, **p < 0.01, ***p < 0.001, and ****p < 0.0001.

## RESULTS

### Identification of molecular subtypes using prognostically relevant PCD genes for MM

**Figure 1:**
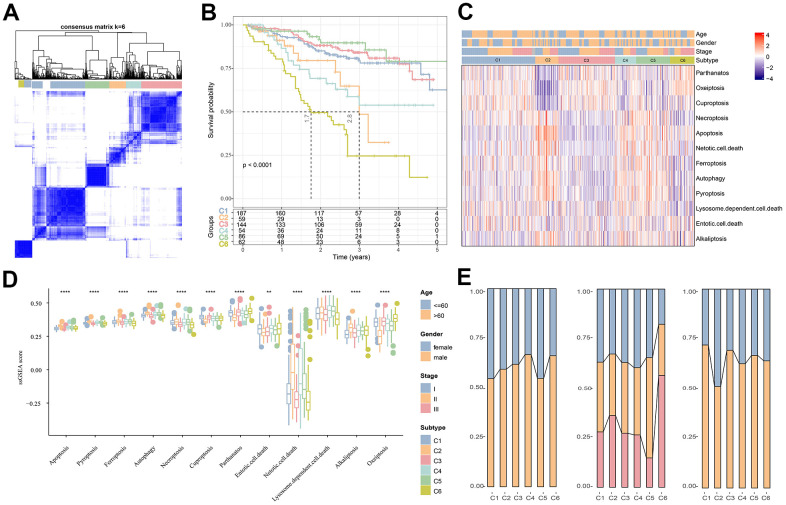
**Cluster analysis of different subtypes of MM based on PCD-related genes.** (**A**) Heatmap of sample clustering in MMRF-COMPASS with a total of k = 6. (**B**) Relationship KM curves of OS prognosis for six molecular subtypes. (**C**) Differences in PCD between molecular subtypes in the MMRF-COMPASS cohort. (**D**) Heat map of statistically significant different PCD levels in different subtypes. (**E**) Clinicopathological characteristics of the six molecular subtypes in the MMRF-COMPASS training cohort. **p < 0.01, ****p < 0.0001.

The association between the expression of PCD-related genes in the MMRF-COMPASS training set cohort patients was analyzed. Here, a total of 434 genes significantly related to the prognosis of MM were filtered (*p*<0,001). Subsequently, according to the expression profiles of the 434 genes, 592 patients in MMRF-COMPASS training cohort were classified by consensus clustering method. From the results of the cumulative distribution function (CDF) Delta area, the CDF downward slope was the smallest at k = 6, which had a more stable clustering result (Supplementary Figure 1). Therefore, the whole cohort was divided into six molecular subtypes (C1, C2, C3, C4, C5, C6) (Figure 1A) with significant prognostic differences (Figure 1B). Overall, MM patients in the C5 subtype had a better prognosis, in contrast, those in the C6 subtype had a lower survival rate. Figure 1C shows the distinct clinical features of MM patients in the six subtypes, and it can be observed that most patients in the C1, C3, and C6 subtypes did not differ significantly in terms of their staging, grading, and age. Meanwhile, the results of differential analysis demonstrated significant differences between different PCD scores across the six molecular subtypes (Figure 1D). Subsequently, analysis on the clinicopathological features between different subtypes in the MMRF-COMPASS training cohort showed a higher grade of the C6 subtype (Figure 1E). Overall, MM prognosis and the expression of PCD-related genes were remarkably different among the six molecular subtypes.

### Association between the molecular subtypes and immunotherapy

**Figure 2:**
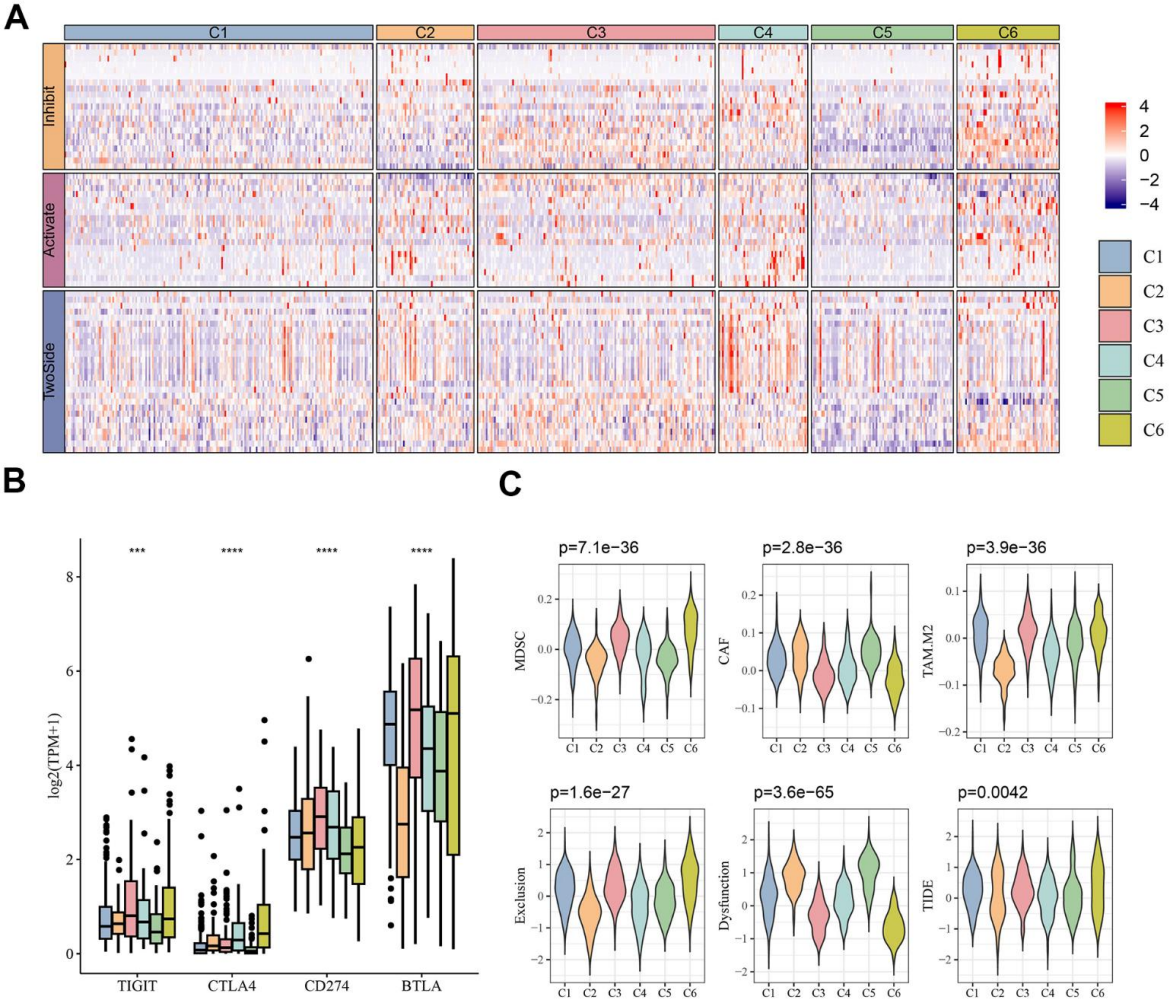
**Immunotherapy analysis.** (**A**) Heatmap of expression of immune checkpoint genes between different molecular subtypes. (**B**) Differential expression of common immune checkpoint genes *TIGIT*, *CTLA4*, *CD274* and *BTLA* between different molecular subtypes. (**C**) Results predicted by TIDE software. ***p < 0.001, ****p < 0.0001.

Considering the efficacy of ICB in cancer immunotherapy, we assessed the differential expression of a representative set of 67 immune checkpoint genes from a previous study [22] among the six molecular subtypes. As shown in Figure 2A, most of the immunosuppressive, immune-activating and TwoSide genes were upregulated in the C4 and C6 subtypes but downregulated in the C1 and C5 subtypes. Additionally, significant differences in the expression of the four immune checkpoint genes (*TIGIT*, *CTLA4*, *CD274* and *BTLA)* were observed among the six molecular subtypes, with *BTLA* having a high expression and TIGIT and *CTLA4* having a low expression (Figure 2B). These results indicated that the expression pattern of the immune checkpoint genes could be considered as a marker for evaluating the immunotherapy responses of MM patients.

Subsequently, it was found that the infiltration abundance of MDSC, CAF and TAM differed significantly across the molecular subtypes, specifically, these three types of cells showed a higher infiltration in C6, C5, and C2, respectively. TIDE analysis also showed that the TIDE score was higher in C6 subtype (Figure 2C). The high exclusion score in the C6 subtype indicated that this subtype evaded the immune system mainly through immune exclusion mechanisms, whereas a high dysfunction score in the C5 subtype indicated that MM patients in this subtype relied on immune cell dysfunction for immune escape. This suggested that different subtypes of MM tumors required different immunotherapeutic strategies to respond effectively.

### Characterization of the pathways for the six molecular subtypes of MM

**Figure 3:**
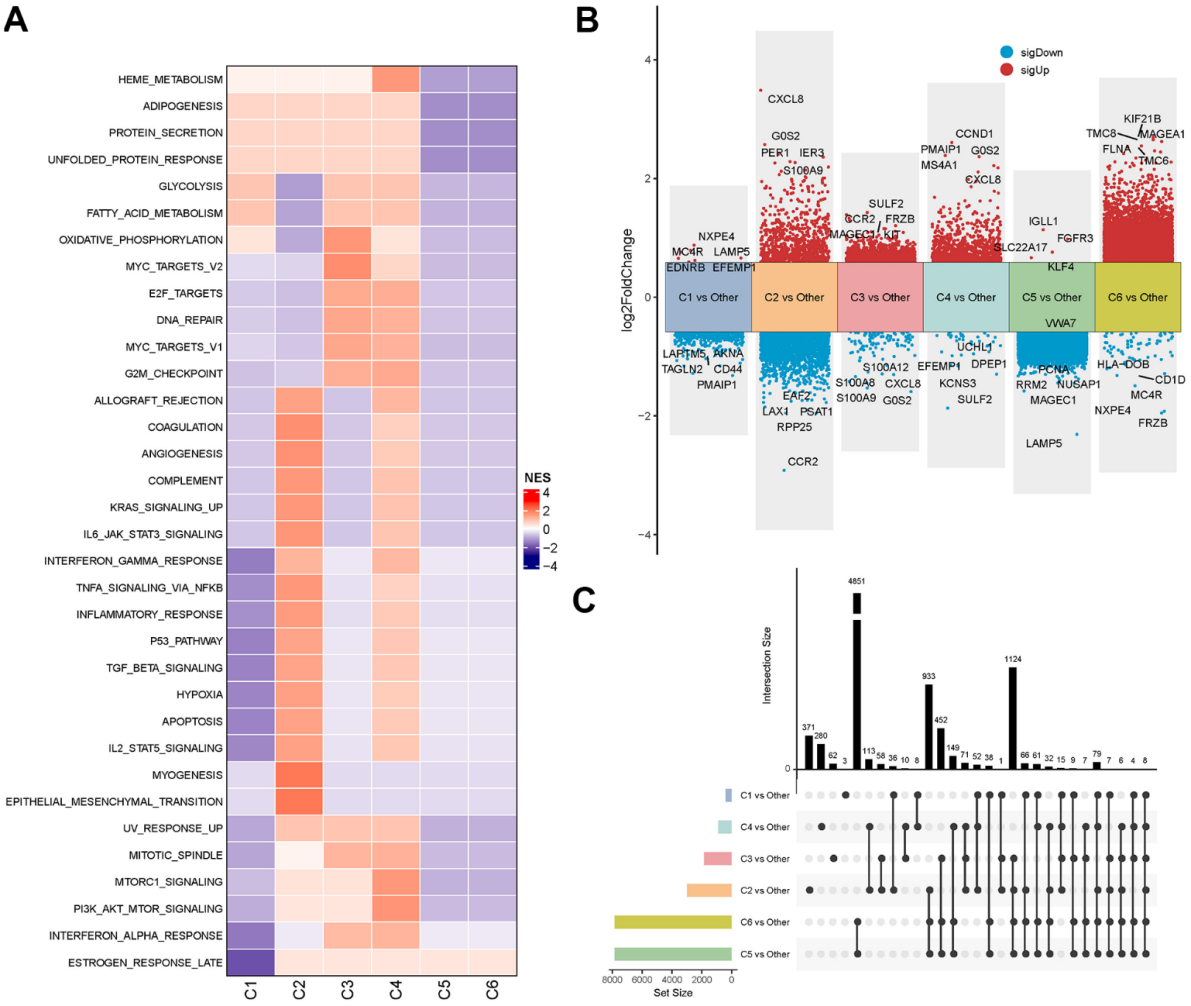
**Signalling and genomic landscapes between different subtypes in the MMRF-COMPASS training cohort.** (**A**) Signaling pathway activities of different molecular subtypes in the MMRF-COMPASS training cohort. Results of differential expression analysis (**B**) and distribution (**C**) of genes between molecular subtypes in the MMRF-COMPASS training cohort.

Using the HALLMARK gene set from the MSigDB database, GSEA was employed to identify differentially activated pathways in the six molecular subtypes of MM, with FDR<0.05 showing a significant enrichment. The analysis revealed that pathways such as oxidative phosphorylation and DNA repair were activated in the C3 subtype and apoptosis-related pathways were more activated in the C2 and C4 subtypes, whereas most signaling pathways appeared to be relatively stable in the C1, C5 and C6 subtypes.

(Figure 3A). In addition, we further identified DEGs by comparing each subtype against other subtypes (e.g., C1 vs. all others, C2 vs. all others, etc.) using the ‘limma’ package under the criteria of FDR < 0.05 and |log2FC| > log2(1.5) (Figure 3B). Moreover, analysis on the distribution of DEGs across these molecular subtypes showed that only eight genes were consistently differentially expressed in all the subtype comparisons (C1 vs. others, C2 vs. others, C3 vs. others, C4 vs. others, C5 vs. others, and C6 vs. others) (Figure 3C). Collectively, these findings indicated that different subtypes of MM were characterized by specific genomic and molecular features, which were closely correlated with MM prognosis.

### Development and validation of a clinical predictive model

**Figure 4:**
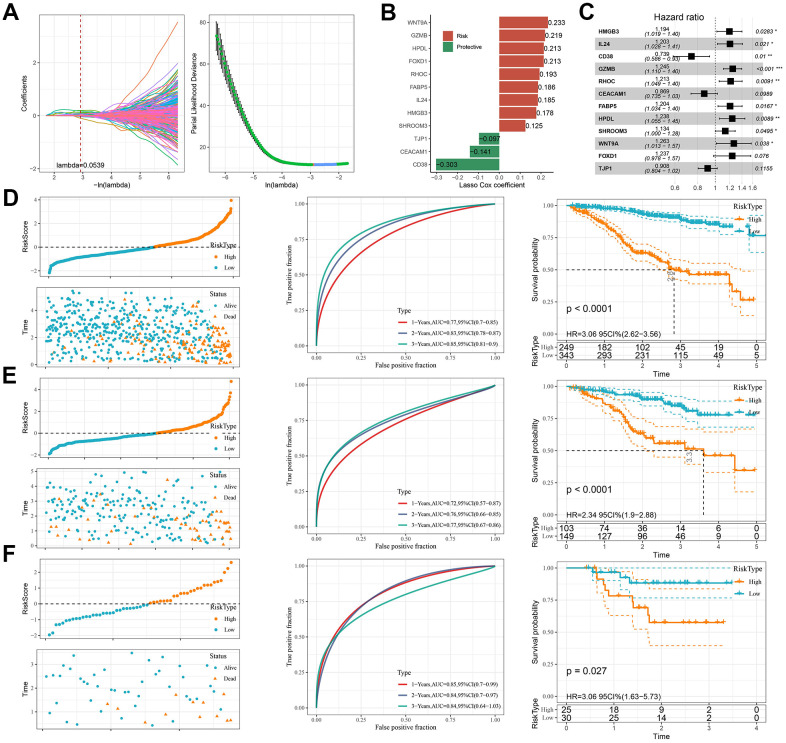
**PCD-based risk model construction and validation.** (**A**) Trajectories of each independent variable with lambda and confidence intervals under lambda. (**B**) Key genes of the prognostic model. (**C**) Forest plot of key genes of the prognostic model. (**D**–**F**) Validation of clinical prognostic models for the MMRF-COMPASS training set cohort, MMRF-COMPASS validation set cohort, and GSE57317 cohort, respectively. They are, from left to right: Risk score, survival time versus survival status and expression of prognostic genes, KM Survival Curve Distribution, and ROC Curve.

Previous analyses identified the DEGs across the six subtypes of MM using the ‘limma’ package. Subsequently, 7035 genes that significantly influenced the prognosis of MM patients were screened by univariate Cox regression analysis and further subjected to LASSO regression using the R package “glmnet” to compress the gene number. As shown in Figure 4A, the number of independent variables whose coefficients tended to zero went up with the gradual increase of lambda. We employed 3-fold cross-validation for the model development and analyzed the confidence intervals under each lambda (Figure 4A). The model was the optimal when lambda=0.0539, under which a total of 24 genes were selected as the target genes for subsequent analysis. Then, using the 24 genes in the LASSO analysis, we conducted stepwise multivariate regression analysis and used the stepAIC method in the MASS package. Starting with the most complex model, one variable was removed iteratively at a time to reduce the AIC. Through this process, we finally identified 12 genes, namely, *HMGB3*, *IL24*, *CD38*, *GZMB*, *RHOC*, *CEACAM1*, *FABP5*, *HPDL*, *SHROOM3*, *WNT9A*, *FOXD1*, and *TJP1* (Figure 4B–4C).

Each patient in the TCGA cohort was assigned with a Risk score calculated by the formula: Risk score=0.178*HMGB3+0.185*IL24-0.303*CD38+0.219*GZMB+0.193*RHOC-0.141*CEACAM1+0.186* *FABP5*+ 0.213*HPDL+ 0.125*SHROOM3+ 0.233*WNT9A+ 0.213*FOXD1-0.097*TJP1.

Next, we compared the survival time of MM patients in different subgroups and found that high-risk patients had shorter survival time than low-risk patients (*p* < 0.0001). Moreover, the AUC values for 1-, 2-, and 3- year survival reached 0.77, 0.83, and 0.85, respectively (Figure 4D). Notably, similar results in the MMRF-COMPASS validation set cohort (*p* < 0.0001) and the GSE57317 cohort (*p* = 0.027) were observed, with high-risk patients showing a significantly worse OS. The AUC values for 1-, 2-, and 3- year survival prediction in the MMRF-COMPASS validation set cohort reached 0.72, 0.76 and 0.77, respectively (*p* < 0.0001, Figure 4E). In GSE57317 cohort, the AUC values for 1-, 2-, and 3- year survival prediction were 0.85, 0.84 and 0.84, respectively (*p* < 0.05, Figure 4F). These results demonstrated robust prognostic predictability of PCD-related genes.

### Establishment and assessment of a nomogram

**Figure 5:**
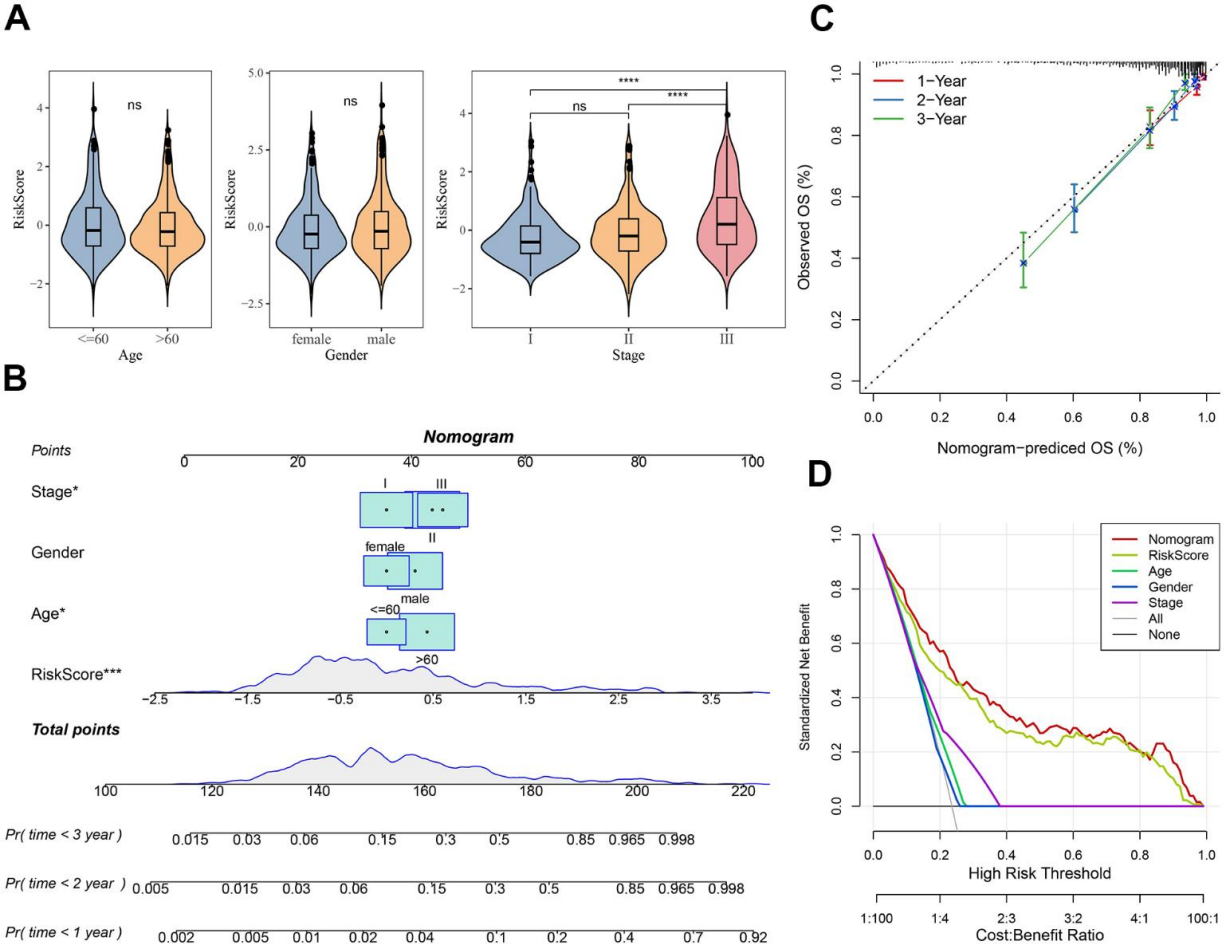
**Nomogram construction for predicting the prognosis of MM patients.** (**A**) Differences in clinicopathological characteristics between risk subgroups in the MMRF-COMPASS training set. (**B**) Risk score combines clinical features to create column-line plots. (**C**) Calibration curves were used to validate the established column line plots. (**D**) Decision curve analysis of column-line plots. Ns represents *p* > 0.05, and ****p < 0.0001.

Subsequently, differences in clinicopathological features between the risk subgroups in the MMRF-COMPASS training set were compared. It was found that gender and age did not differ significantly between the two risk groups, whereas the Risk score increased as the clinical grade advanced (Figure 5A). We further performed multivariate Cox regression analysis combining stage, age, gender, and the Risk score to establish a nomogram to better evaluate OS for MM patients. As shown in Figure 5B, the Risk score showed the greatest impact on the OS prediction in MM, followed by age and stage. Further, the calibration curves demonstrated that the 1-, 2-, and 3-year prediction calibration curves were close to the standard ones, suggesting that the model had a high prediction accuracy (Figure 5C). Interestingly, the accuracy of our risk model further assessed by the decision curve analysis (DCA) also performed well in assessing MM prognosis. (Figure 5D).

### Enrichment pathway analysis and drug sensitivity assessment for MM patients in different risk groups

**Figure 6:**
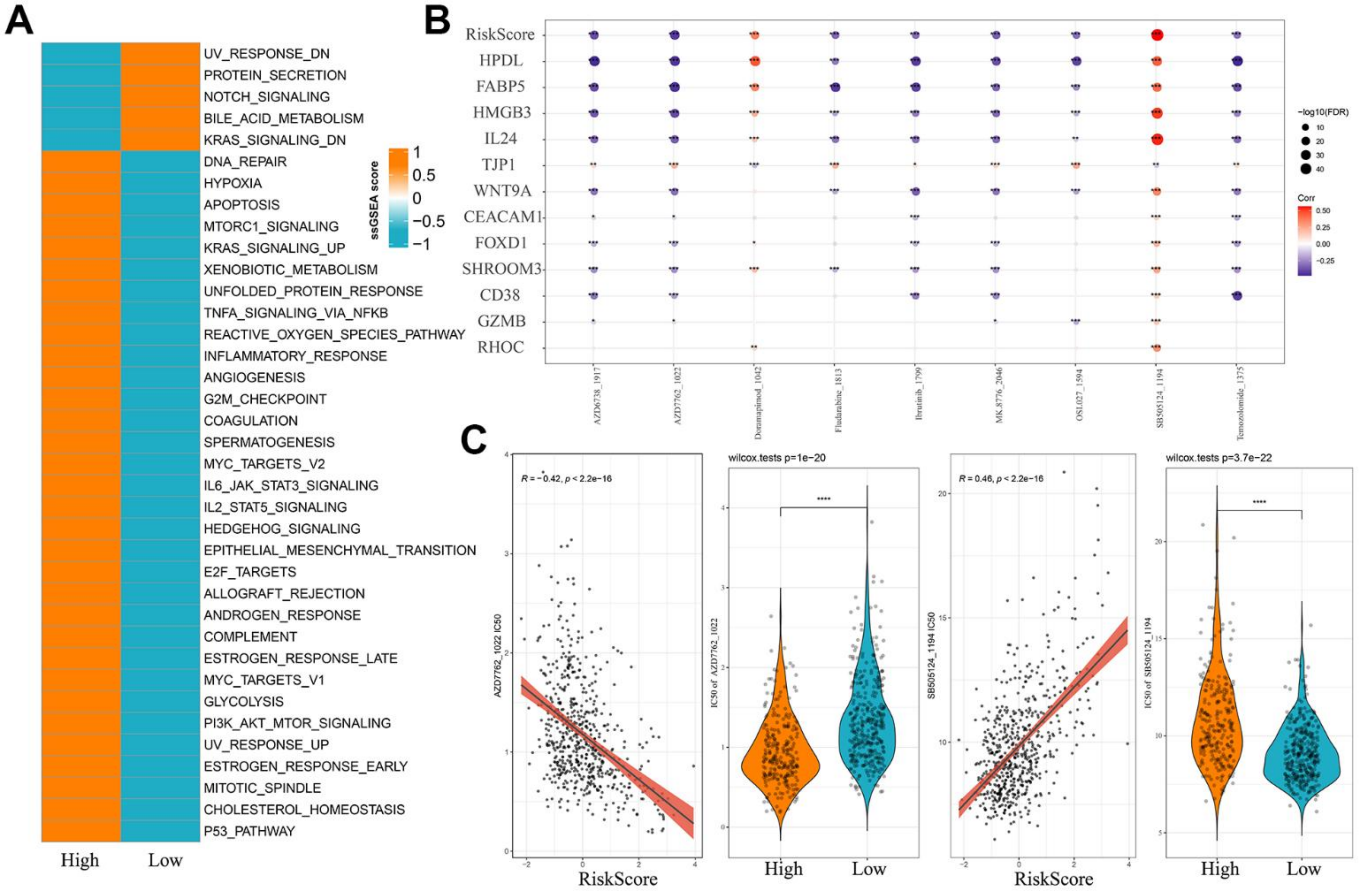
**Risk models and pathway characteristics and drug sensitivity differ between patients in different MM risk groups.** (**A**) HALLMARK pathway differences between high and low-risk subgroups. (**B**) Bubble plots of Risk score in the MMRF-COMPASS training set cohort and expression of key genes in the model versus drug IC_50_ values, size and color indicate the strength of the association. (**C**) Comparison of IC_50_ scores versus drugs between high and low-risk groups. *p < 0.05, **p < 0.01, ***p < 0.001, and ****p < 0.0001.

Subsequently, we calculated ssGSEA scores for each sample based on the HALLMARK Pathway and used Wilcoxon rank-sum test to identify differential pathways between the two risk groups of MM. Pathways such as hypoxia, angiogenesis and apoptosis were notably activated in the high-risk group, whereas pathways including NOTCH_SIGNALING and KRAS_SIGNALING_DN were more activated in the low-risk group (Figure 6A). After calculating the IC_50_ values for each drug in the MMRF-COMPASS training set cohort, a significant correlation (FDR<0.05 and |cor|>0.3) between nine drugs and the Risk score was detected. Analysis on the correlation between the Risk score, the expression of key model genes and the IC_50_ values of the drugs revealed that MM patients in the high-risk group had a higher IC_50_ values for the drug SB505124_1194 (Figure 6B, 6C), suggesting that MM patients with higher Risk score value might be resistant to SB505124_1194 (*p*=3.7e-22). Similarly, patients in the low-risk group may be resistant to AZD7762_1022 (*p*=1e-20). Thus, SB505124_1194 and AZD7762_1022 were considered as valid references for evaluating chemotherapy resistance in MM patients.

## DISCUSSION

Due to drug resistance and surgical recurrence, MM as the second most common hematological malignancy is largely incurable [33]. Study confirmed that PCD is crucially involved in carcinogenesis, immunological infiltration, progression, prognosis, and treatment effectiveness of cancers [34, 35]. For example, Zou et al. [12] developed a PCD signature based on immune checkpoint genes to evaluate the prognosis and drug sensitivity for patients with triple-negative breast cancer. Similarly, Qin et al. [36] discovered 16 PCD genes that are highly effective in predicting the prognosis and immunotherapy response for patients with oesophageal squamous cell carcinoma. Additionally, a significant correlation between PCD and immune features, including immune cell infiltration and the expressions of immune checkpoint molecules, has also been found in lung adenocarcinoma [37]. At present, the study of PCD in MM is largely limited to individual form of PCD, such as apoptosis [38], ferroptosis [39], autophagy [40] and cuproptosis [41]. However, different PCD forms are not necessarily mutually exclusive. Numerous redundancies and crosstalk have been observed in signaling pathways that regulate these patterns of cell death, suggesting that they may work together to influence tumorigenesis and tumor progression [42]. Therefore, using predictive features that integrate information from multiple PCDs may better characterize tumor status. This study analyzed the integrated landscape of PCD in the context of translational medicine, aiming to improve the understanding of the role of PCD in MM.

The TME and its components provide the foundation for the proliferation and survival of malignant cells [43]. The TME is also involved in checkpoint blockade immunotherapy and immunosuppression [44]. According to previous reports, immunosuppressive cell infiltration in the TME is a key marker of the immune microenvironment in tumors, significantly influencing the development of malignancies. Pro-tumourigenic immune cells are attracted by CAF to prevent pro-tumourigenic CD8^+^T cell infiltration [45]. Furthermore, in addition to impeding the invasion of immune cells, MDSC in the TME can directly bind to immune checkpoint receptors on tumor cells, blocking the oncogenic effects of T cells [46]. Similarly, TAM infiltration in TME is related to treatment resistance, metastasis, and immunosuppression in the majority of malignancies [47]. It has been demonstrated that immunological failure in MM patients is associated with the infiltration of immunosuppressive cells, and that the immunosuppressive mediators of these cells are correlated with the prognosis of patients [48, 49]. In the present study, high MDSC infiltration in the C6 subtype may have a suppressive effect on immune cell functions and this was correlated with high rejection scores in the subtype, suggesting that MDSC may evade the immune system mainly through immune rejection mechanisms. Similarly, high CAF infiltration in the C5 subtype was associated with a high dysfunction score, indicating that CAF cells may rely on immune cell dysfunction for immune escape. In addition, macrophage infiltration could also induce immune suppression. Patients with higher TIDE scores have shown a higher likelihood of immune escape and less active response to immune checkpoint inhibitors (ICI) therapy [23]. Overall, TAM, MDSC and CAF cell infiltration in the TME may be the main cause of immune escape in MM patients.

Immune checkpoints could preserve the balance in the body and inhibit abnormal immune response activation. Tumor cells, however, avoid immune response by taking advantage of this feature of immune checkpoint molecules [50]. Immune checkpoint blockade (ICB) therapy has attracted much research interest and demonstrated promising outcomes in cancer treatment [51]. The immunoglobulin-associated receptor family (*TIGIT*, *CTLA4*, *CD274*, and *BTLA*) have inhibitory effects on T cell function and have been used as a part of immunomodulatory strategy for treating cancers [52]. Study reported that high-expressed *CTLA4* and *CD274* in head and neck squamous cell carcinoma (HNSCC) may cause immune dysfunction in the patients [53]. In addition, Hong et al. also found a positive link between the expression level of *BTLA* and that of *TIGIT* in renal cell carcinoma, showing the potential to be considered as a pair of targets in the immunotherapy for the tumor [54]. It was found that dysregulated expression of immune checkpoints may account for a lower clinical response to immune checkpoint therapy in MM [55]. Here, we found significant differences in the expression of *TIGIT*, *CTLA4*, *CD274* and *BTLA* among all the six molecular subtypes. Notably, *BTLA* had higher expression levels in these molecular subtypes, suggesting that immune checkpoint therapy blocking BTLA may have better efficacy to MM patients in different subtypes.

To further improve the clinical applicability, we identified 12 PCD-related genes that affected the prognosis of MM using stepwise regression analysis, including *HMGB3*, *IL24*, *CD38*, *GZMB*, *RHOC*, *CEACAM1*, *FABP5*, *HPDL*, *SHROOM3*, *WNT9A*, *FOXD1*, and *TJP1*. *HMGB3* could regulate breast cancer cell autophagy and apoptosis, promoting cell migration, invasion and metastatic potential [56]. *IL24* inhibits MM cell tumor growth by inducing tumor cell autophagy, thus suppressing MM cell tumor growth [57]. The transmembrane glycoprotein *CD38* mediates T-cell activation and has an immunomodulatory effect on the TME in MM [58]. Although, CD38 expression is commonly increased in MM, it is present as a tumor suppressor in HNSCC [59]. This phenomenon may be related to the drug resistance of the samples selected in this study, which requires further validation. *GZMB* has been reported to serve as an immune response regulator in the immune activation of artificial pluripotent stem cells in MM [60]. Li et al. [61] observed that *GZMB* hinders immune evasion by inducing pyroptosis and apoptosis in acute myeloid leukemia (AML) cells. In addition, *RHOC*, a ferroptosis and cuproptosis-related gene that affects the prognosis of AML, has a strong ability in predicting the OS of the cancer [62]. *RHOC* can promote tumor growth and induce tumor angiogenesis in MM [63]. In addition, *CEACAM1* plays an oncogenic role in MM by inhibiting tumor cell proliferation, invasion and migration, and inducing apoptosis [64]. The lipid chaperone protein *FABP5* promotes cell proliferation, inhibits apoptosis and enhances chemotherapy resistance in MM patients [4, 65]. High expression of *HPDL* in pancreatic ductal adenocarcinoma is predictive of poorer prognosis and immunosuppression in the patients [66]. In addition, bioinformatics analysis revealed that *SHROOM3* is a strong predictor of prognosis, immune activity, and treatment response of clear cell renal cell carcinoma [67]. Previous research verified that the mRNA expression level of *WNT9A* is significantly associated with the biochemical recurrence of prostate cancer [68]. *FOXD1* could be able to promote cell proliferation and inhibit apoptosis by regulating polo-like kinase 2 in colorectal cancer [69]. Another study reported that *TJP1*, whose expression is low in MM, inhibits tumor metastasis by increasing the adhesion of MM cells to bone marrow stroma [70]. These results suggested that most of these genes were associated with the development of one or more PCD forms in tumors, and that their different roles in tumors may be related to the TME. In accordance with the available analyses, these genes were largely linked to cell invasion and metastasis, immune infiltration and therapeutic response, which was of important value in the prognostic prediction in MM.

Subsequently, we identified differential pathways between high- and low-risk groups, and found that pathways such as hypoxia and angiogenesis, which were closely related to tumor development and progression, were remarkably activated in the high-risk group [71]. The activation of the apoptotic pathway once again proved the anti-tumor effect of *CEACAM1* in MM [64]. In the low-risk group, pathways such as NOTCH_SIGNALING and KRAS_SIGNALING_DN were more activated, indicating that the bone marrow microenvironment of low-risk MM patients may be an ideal microenvironment for the proliferation and migration of MM cells [72–74]. These results suggested that differences in tumor behaviors in different subgroups of MM may be a result of aberrant activation of different genes and pathways, which was a crucial cause leading to tumor progression and a key clinical target. The therapeutic efficacy of post-surgical adjuvant chemotherapy in treating most tumors has been widely recognized [75]. However, the heterogeneity of the TME may cause resistance and different responses to the therapy in different patients [8]. Similarly, improving drug resistance in MM patients could contribute to a better prognosis of the patients. Therefore, we developed a risk model with the sensitivity to several most commonly used drugs in MM therapies. Higher IC_50_ values of SB505124_1194 in the high-risk group may explain the unfavorable prognostic outcomes in this group. The anticancer sensitivity of the drug predicted by ferroptosis-related genes has also been verified in AML [76]. AZD7762 could enhance tumor cell sensitivity to DNA damage and cisplatin-induced apoptosis in osteosarcoma cells [77]. Considering the fact that low-risk MM patients may be resistant to AZD7762_1022, it can be speculated that MM patients could respond to most PCD-related chemotherapeutic agents. These findings supported that the Risk score developed with the PCD genes contributed to the clinical management of MM patients.

However, there were certain limitations in this study to be noted. Firstly, bias may be unavoidably caused by the retrospective recruitment of patients. Secondly, additional experimental research is required to analyze the biological roles of some PCD genes that have not been investigated in MM cells. Furthermore, this study only assessed the sensitivity of MM to two different types of medications applying bioinformatics analysis, therefore tissue tests are needed to confirm the validity of the current results. Finally, multicenter randomized controlled studies with large sample sizes and follow-up data are also encouraged to be carried out for further validation.

## CONCLUSIONS

In this study, six molecular subtypes of MM were identified using PCD genes, which can be employed to characterize different prognostic and immune states of MM patients. In addition, a robust 12-gene Risk score model developed based on the differential genes were independent of clinicopathological characteristics and showed stable prediction performance in both independent datasets. Moreover, the model could be applied to assess the sensitivity of MM patients to anticancer drugs. This helped to better understand the mechanism through which PCD influenced the progression of MM and also provided a theoretical reference for the clinical targeted therapy of MM.

## Supplementary Material

Supplementary Figure 1
