# Supplementary material for: Comprehensive genomic characterization of programmed cell death-related genes to predict drug resistance and prognosis for patients with multiple myeloma
Source: Aging (Albany NY). 2025 Apr 1;17(4):1043–59. doi: 10.18632/aging.206234 (PMC12074814; doi:10.18632/aging.206234)
Supplement: Supplementary Figure 1 [file aging-17-206234-s001.pdf]

SUPPLEMENTARY FIGURE

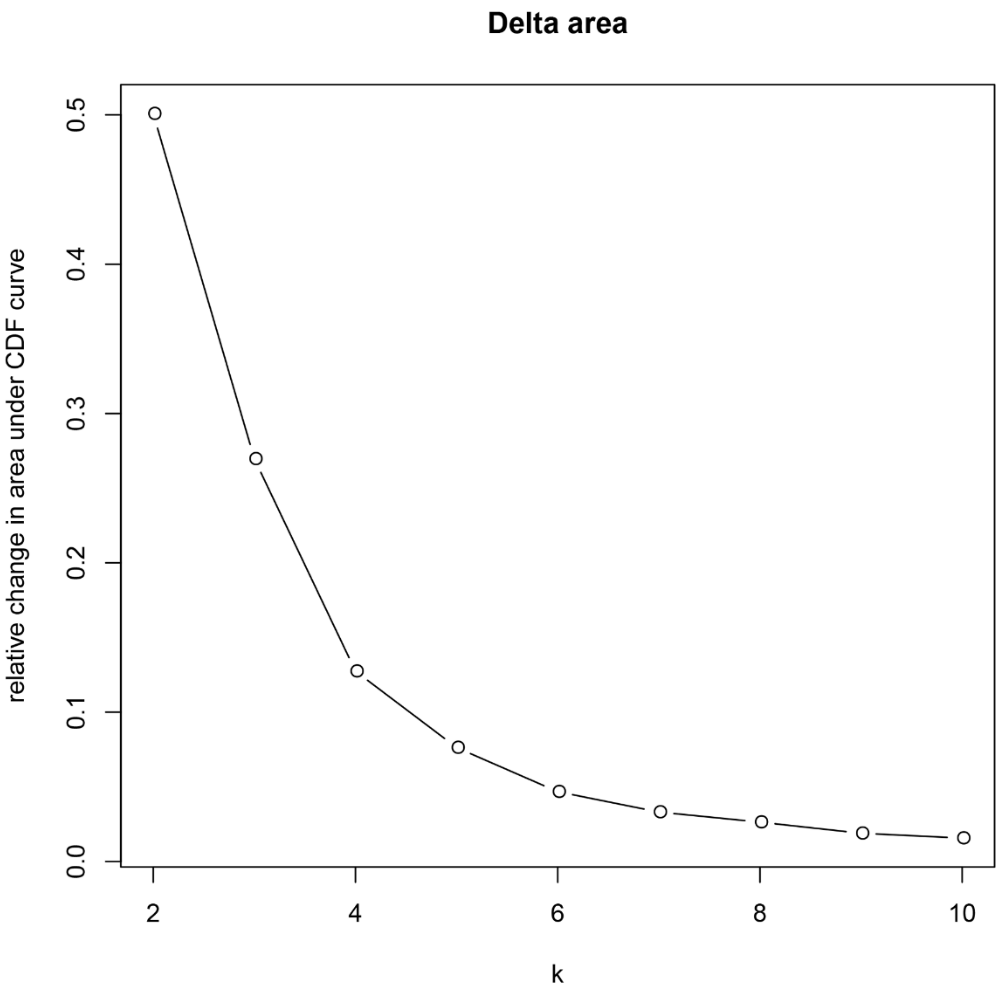

Supplementary Figure 1. Formulaic cluster analysis of MM prognosis-associated PCD genes. (S1) Horizontal coordinates indicate the number of categories k and vertical coordinates indicate the relative change in area under the CDF curve.
